# Multifocal micronodular pneumocyte hyperplasia lacking typical clinical features of the tuberous sclerosis complex: a case report and literature review

**DOI:** 10.1186/s12890-022-01849-8

**Published:** 2022-03-05

**Authors:** Shan Li, Chaojie Wu, Qiyun Ma, Xueqin Chen, Wei Zhang, Xiao Li, Mao Huang, Ningfei Ji

**Affiliations:** 1 Department of Respiratory and Critical Care Medicine, the First Affiliated Hospital of Nanjing Medical University, Nanjing, 210029 China; 2 Department of Radiology, the First Affiliated Hospital of Nanjing Medical University, Nanjing, 210029 China; 3 Department of Pathology, the First Affiliated Hospital of Nanjing Medical University, Nanjing, 210029 China

**Keywords:** Multifocal micronodular pneumocyte hyperplasia, Tuberous sclerosis complex, Lymphangioleiomyomatosis, Genetic sequencing, Case report

## Abstract

**Background:**

Multifocal micronodular pneumocyte hyperplasia (MMPH) is a rare pulmonary manifestation of the tuberous sclerosis complex (TSC) with distinctive histological characteristics. Most case reports of MMPH associated with TSC usually have a history and typical clinical features (seizures, mental retardation, and skin lesions) of TSC. We present a peculiar asymptomatic MMPH case that lacked the history and typical clinical features of TSC.

**Case presentation:**

A 56-year-old man was referred to our hospital with bilateral ground-glass opacities (GGOs) on chest computed tomography (CT) lasting 8 months, with no complaint of any discomfort. Because of the lack of clinical manifestations, the diagnosis of MMPH and TSC was confirmed by lung biopsy histopathology and gene sequencing of nonsense mutations in the TSC1 gene. Considering the relevant literature review and that the prognosis of most patients with MMPH is generally stable, no special treatment was given. We followed up with the patient for three years after discharge, and the clinical manifestations and imaging features of the patient were stable.

**Conclusion:**

To our best knowledge, this is the first case of MMPH lacking typical clinical manifestations of TSC confirmed by histopathology combined with gene sequencing. MMPH should be considered as one of the differential diagnoses of multiple GGOs in the lung even when the findings of TSC are not recognized.

## Background

Multifocal micronodular pneumocyte hyperplasia (MMPH) is a rare pulmonary manifestation of the tuberous sclerosis complex (TSC) and an autosomal dominant disorder characterized by seizures, facial angiofibroma and mental retardation [1]. Some previous studies have reported that MMPH is common in patients with TSC, and its prevalence rate is approximately 40–60%[2, 3]. However, whether the diagnosis of MMPH is only preliminarily confirmed by chest computed tomography images without histopathology in these studies remains controversial. Because most MMPHs show no respiratory symptoms, the diagnosis is mainly based on histopathology. The histological characteristics of MMPH are multifocal and small nodular hyperplasias of the alveolar epithelium, accompanied by increased elastic fibers in the alveolar septum within the nodule [4, 5]. However, distinguishing MMPH from atypical adenomatous hyperplasia (AAH), adenocarcinoma in situ (AIS) and minimally invasive adenocarcinoma (MIA) in histopathological manifestations is challenging [4]. Most of the previously reported MMPH cases were combined with typical clinical manifestations of TSC, except for an MMPH case without typical TSC clinical characteristics reported in Japan in 2011 [6]. In that case, the diagnosis of MMPH was confirmed by histopathology, no further genetic sequencing of TSC was performed. In contrast, we present the first case of MMPH lacking typical clinical characteristics of TSC diagnosed with pathological examination and genetic sequencing.

## Case presentation

A 53-year-old man without respiratory symptoms had undergone chest computed tomography (CT) during a routine physical examination 8 months prior, revealing multiple ground glass opacities (GGOs) in the bilateral lungs. Considering the endemicity of tuberculosis in China he was empirically treated with isoniazid, rifampicin, pyrazinamide and ethambutol (HRZE) at a local hospital. However, no radiologic improvement occurred, and bronchoscopy showed no special abnormalities after 6 months of anti-tuberculosis treatment. Additionally, bronchoscopy showed no abnormalities except for left upper lobe bronchial mucosa congestion at the local hospital. After one month of anti-infective treatment with roxithromycin at another hospital, the radiological changes were still not improved. Thus, the patient was referred to our department for further diagnosis and treatment. In addition to a history of hypertension, he had neither a history of other diseases nor a family history of diseases. The patient worked in a woolen mill with a history of dust exposure. No special abnormality was found on physical examination.

**Fig. 1:**
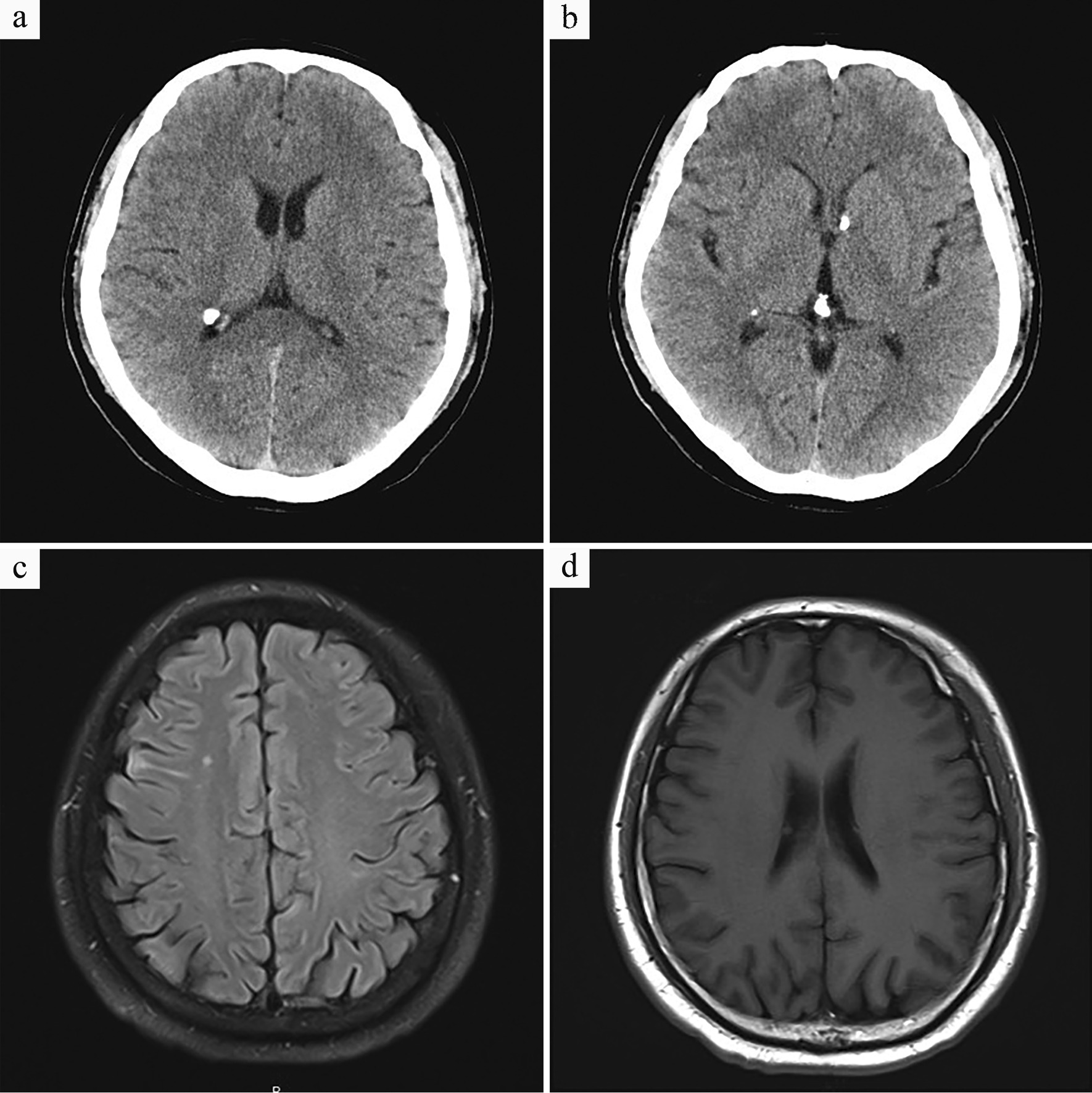
Imaging findings of the skull. **a**, **b** Multiple subependymal calcified nodules in bilateral ventricles. **c** Multiple nodular abnormal signals on the right side of the paraventricular ependyma are shown on T1WI. **d** Patchy abnormally high density in the subcortical area of the right frontal lobe is shown on T2WI

**Fig. 2:**
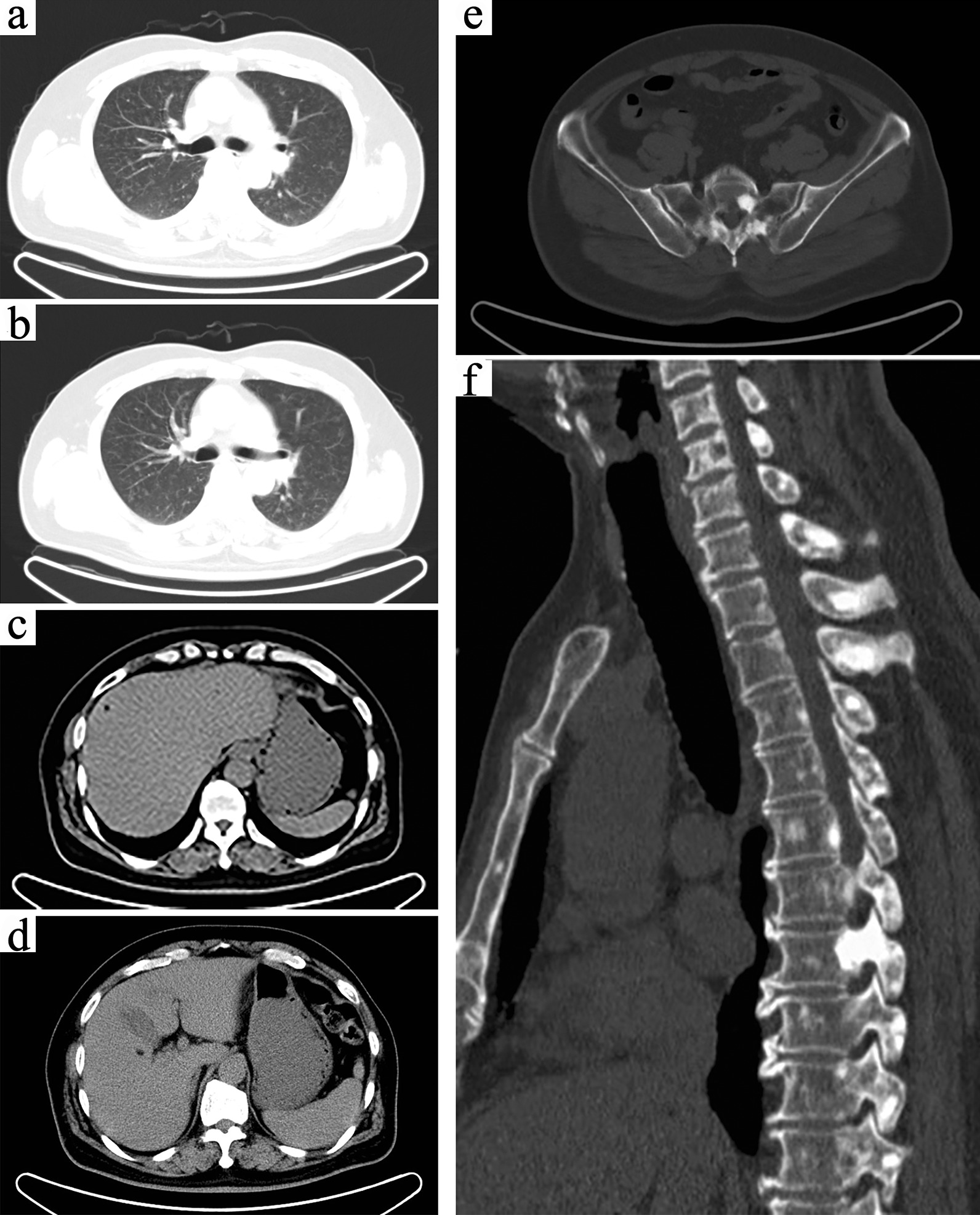
Images of the lungs, liver and bones in the PET-CT of the patient. **a**, **b** Diffuse ground glass density nodules throughout the lung fields. **c**, **d** Multiple low- density shadows containing fat density in the liver. **e**, **f** The bone mineral density of the thoracic vertebra and its appendages, sternum and bilateral ribs increased, showing hyperplastic and sclerosing changes

Positron emission computed tomography (PET-CT) was performed, which showed multiple subependymal calcified nodules in bilateral ventricles (Fig. 1a, b); diffuse ground-glass density nodules throughout the lung fields, particularly in the middle and upper lungs, with no significant increase in FDG metabolism (Fig. 2a, b); multiple low-density shadows containing fat density in the liver without an increase in FDG metabolism (Fig. 2c, d); multiple nodular dense shadows in the bone with some FDG metabolism slightly increased (Fig. 2e, f). According to the guidance of the radiology department, the above lesions were considered multiple organ involvement of TSC (e.g., brain, lung, liver, and bone), in which bilateral lung lesions were consistent with the imaging manifestations of MMPH. Additionally, the examination also revealed multiple cysts in both kidneys.

### Lung biopsy

**Fig. 3:**
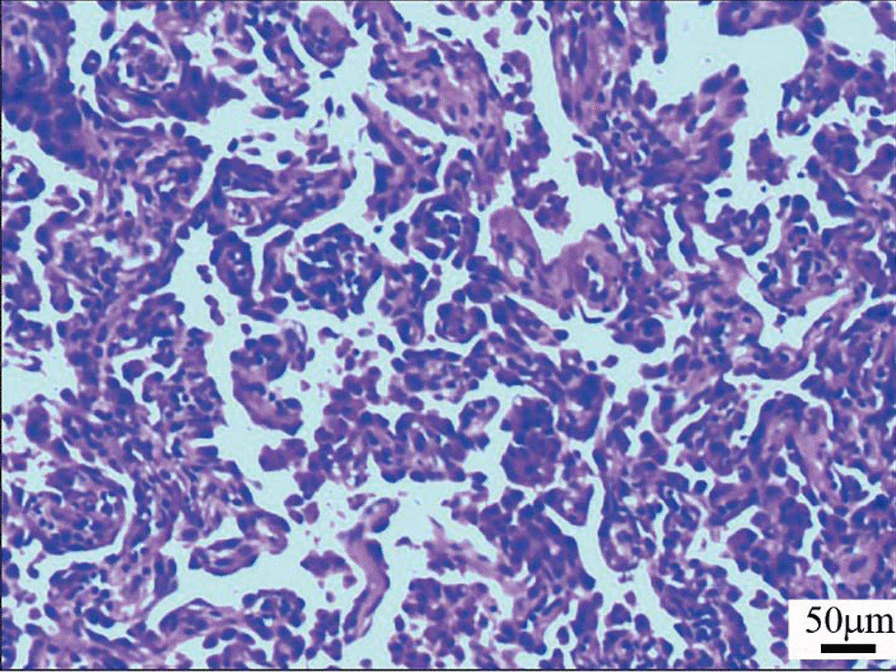
Histopathological images: the alveolar epithelium in the diseased region proliferated actively, and many tissue cells gathered in the alveolar cavity (hematoxylin and eosin staining, × 100). Microscope: Nikon Eclipse 80i; Microscope camera: LG300; Acquisition software: Toup View 3.7

**Fig. 4:**
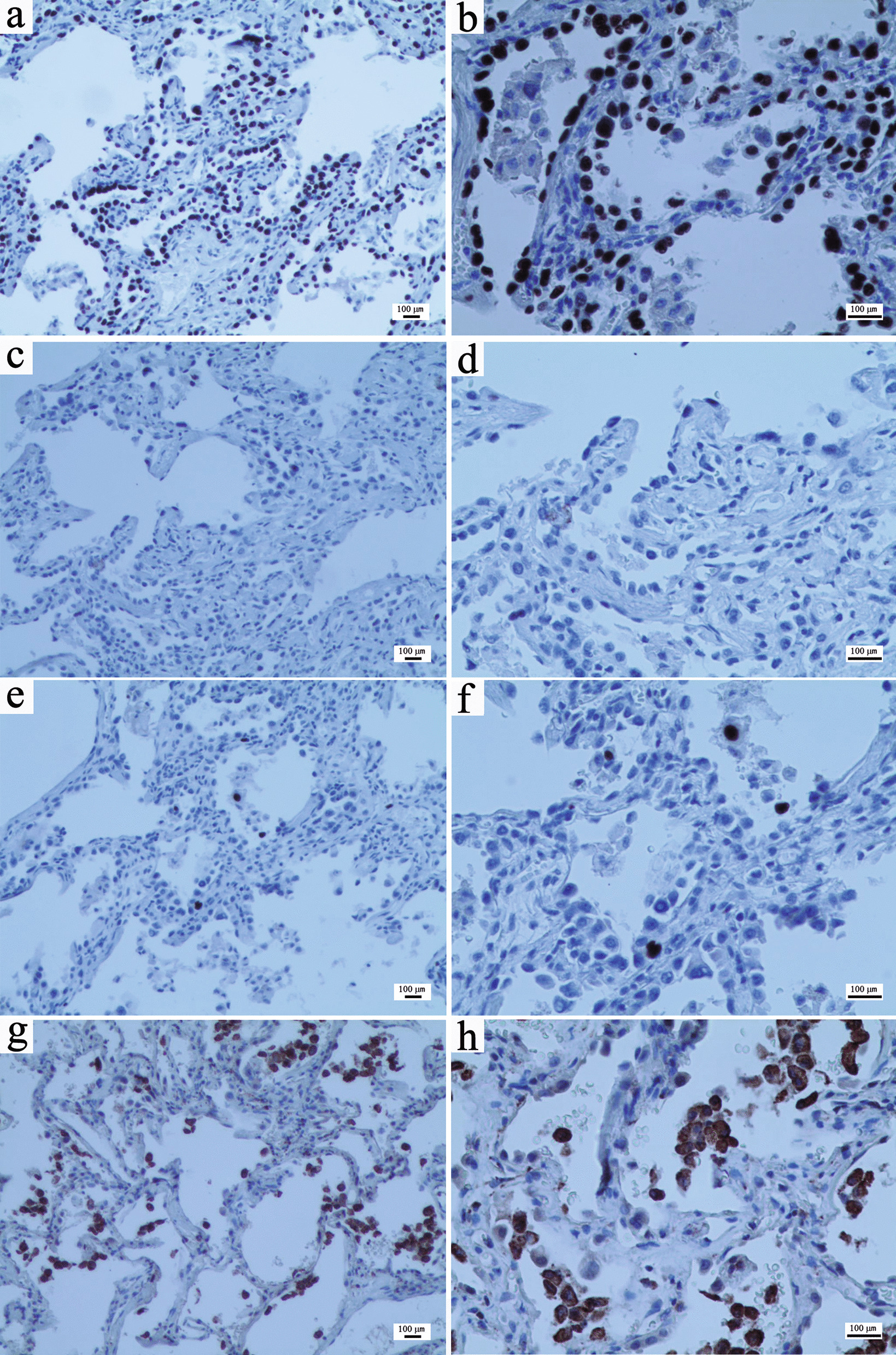
Immunohistochemistry of the biopsy specimen. **a** TTF-1 alveolar epithelium cells ( +) (magnification, × 100). **b** TTF-1 alveolar epithelial cells ( +) (magnification, × 200). **c** p53 scattered alveolar epithelial cells (+ , wild type) (magnification, × 100). **d** p53 scattered alveolar epithelial cells (+ , wild type) (magnification, × 200). **e** Ki-67 ( +) in individual alveolar epithelial cells, with a positive index less than 2% (magnification, × 100). **f** Ki-67 ( +) in individual alveolar epithelial cells (magnification, × 200). **g** CD68 (+ +) tissue cells of the alveolar cavity (magnification, × 100). **h** CD68 (+ +) tissue cells of the alveolar cavity (magnification, × 200). Microscope: Nikon Eclipse 80i; Microscope camera: LG300; Acquisition software: Toup View 3.7

To further explore the nature of the nodules in the bilateral lungs, a lung biopsy was performed with the consent of the family. Two wedge-shaped resection specimens of the right upper lung and right lower lung were sent for examination during the operation. In the section of the specimen, more than 10 scattered gray-white and gray-yellow nodules were observed with the naked eyes, the diameter of which was 0.2–0.7 cm. Microscopically, the alveolar epithelium in the diseased region proliferated actively. Additionally, some of them grew like spikes attached to the wall, many tissue cells gathered in the alveolar cavity, and widening of the alveolar space was observed in some nodules, with fibrous tissue hyperplasia and scattered lymphocyte infiltration (Fig. 3). Immunohistochemistry showed TTF-1 (+), p53 (scattered+, wild type), ki-67 (individual+), and CD68 (tissue cells in alveolar cavity++) in the alveolar epithelium, elastic fiber staining showed elastic fiber hyperplasia in some nodules, and the lesion did not involve the pulmonary membrane (Fig. 4).

### Differential diagnosis

**Fig. 5:**
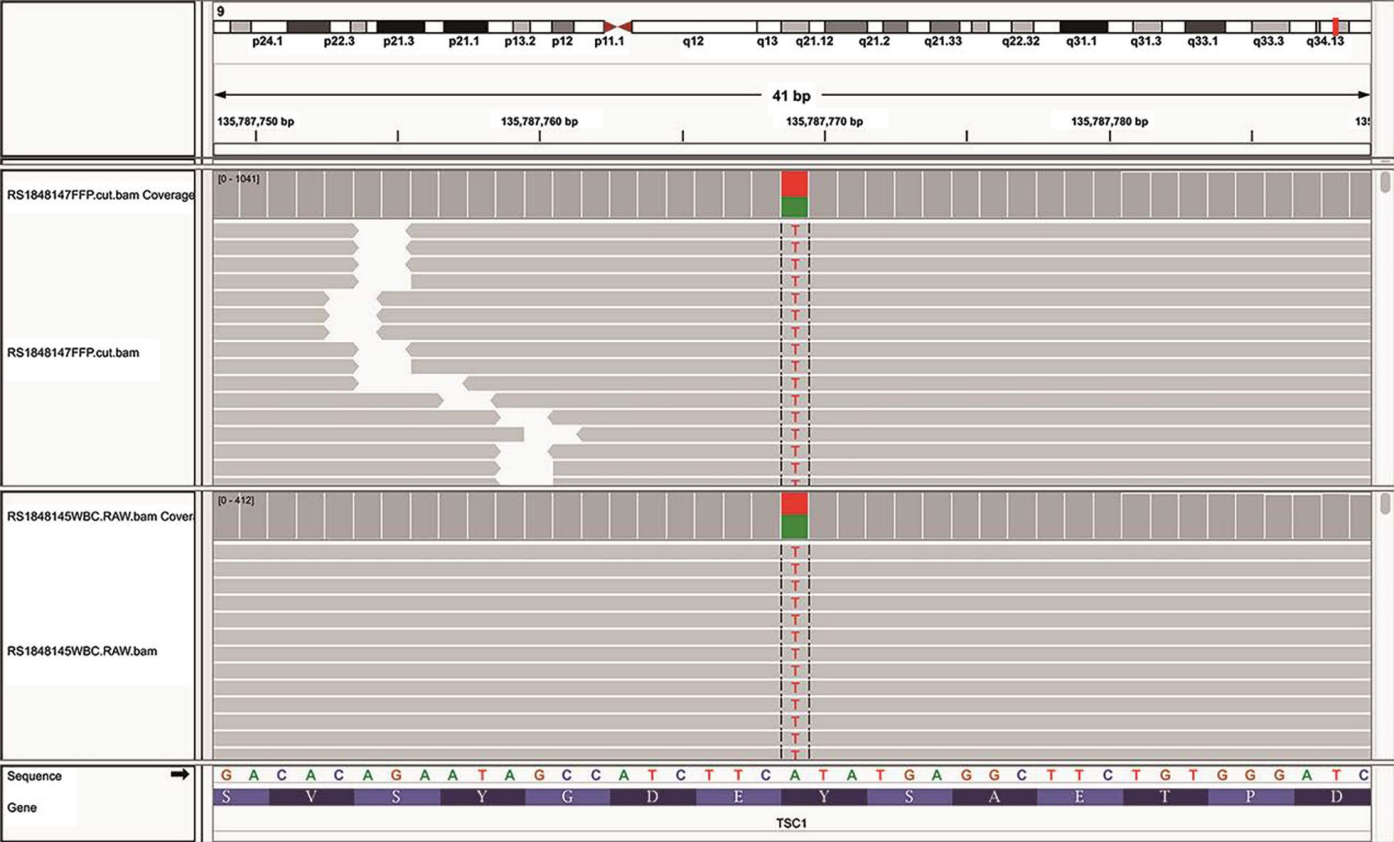
TSC gene sequencing showed that the 271st amino acid of the TSC1 gene mutates from tyrosine to terminator, and the premature terminator may cause nonsense-mediated mRNA degradation, resulting in loss of protein expression

Because MMPH is extremely rare and its cell morphology is difficult to differentiate from lung AAH, AIS and even MIA, further genetic tests were performed to identify MMPH. Molecular pathology showed no mutations in ALK, ROS1, KRAS, NRAS, BRAF, PIK3CA, HER2, RET, or EGFR. The pathological results were consistent with the histological characteristics of MMPH. According to the relevant literature [7], MMPH is rare and occurs mostly in patients with TSC. Additionally, most patients have typical clinical features of TSC; however, the clinical manifestations of TSC in this patient were insufficient, and no typical Vogt’s triad was observed (epilepsy, mental retardation, sebaceous adenoma). According to the latest diagnostic criteria of TSC in 2012 [2], the patient met 1 major criterion (subependymal nodules) and 1 minor criterion (multiple renal cysts) and met only the diagnostic criteria of a "possible diagnosis" in terms of the clinical diagnosis. To further confirm the diagnosis of TSC, TSC gene sequencing was performed. The nonsense mutation in exon 9 of the TSC1 gene (c.813T>A) resulted in the mutation of the 271st amino acid from tyrosine to terminator, and the premature terminator may cause nonsense-mediated mRNA degradation (NMD), resulting in the loss of protein expression (Fig. 5). Accordingly, the patient was eventually diagnosed with MMPH secondary to TSC. Surprisingly, most patients with sporadic TSC had positive TSC2, and those with a family history of TSC had positive TSC1. However, the patient with no family history of TSC tested positive for TSC1. Because of economic conditions and time coordination, the relatives refused to perform TSC gene sequencing in the family.

### Post-discharge follow-up

**Fig. 6:**
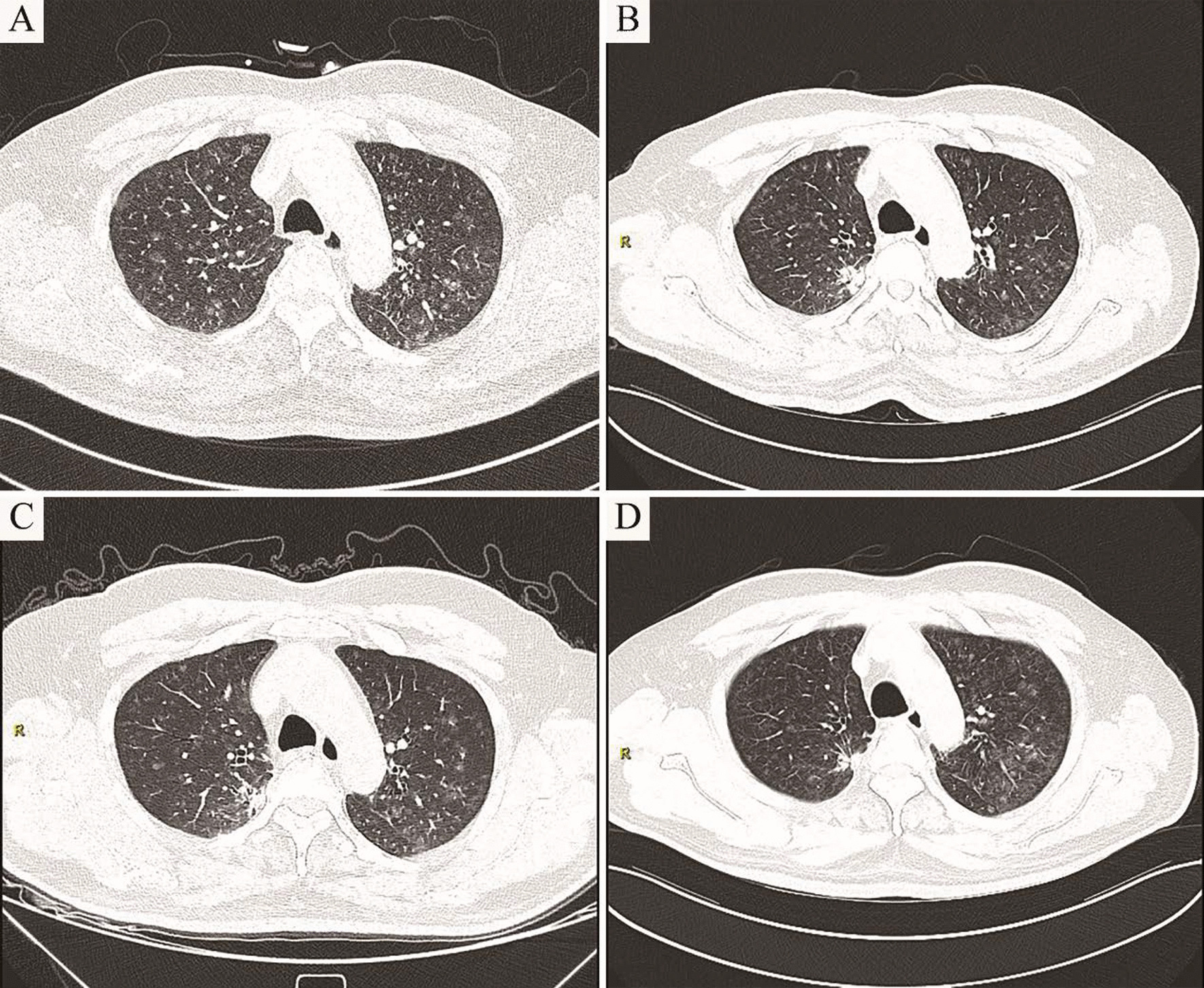
Imaging changes on Computed tomography. **a** HRCT showed multiple randomly distributed ground glass nodules in the center of the lobules throughout the lung fields, particularly in the upper lungs (2018-05-23). **b** HRCT after three months of follow up showed no significant change in GGOs in the bilateral lungs (2018-09-03). **c** HRCT after follow up for half a year revealed no significant change in GGOs (2018-12-13). **d** HRCT after follow up for a year revealed no significant change in GGOs (2019-07-08)

Next, we followed up with the patient for up to three years. During this period, the patient showed no respiratory symptoms. Additionally, no significant change was found in the diffuse nodules of the bilateral lungs within 1 year (Fig. 6). Cranial imaging showed multiple nodular low signals in the bilateral ventricular walls, consistent with tuberous sclerosis (Fig. 1c, d). In summary, the imaging signs tended to be stable during the one-year follow-up course of the patient.

## Discussion and conclusion

MMPH, first described by Popper et al. in 1991 [8], is a manifestation of pulmonary hamartoma in TSC. MMPH often co-occurs with TSC; however, individual cases have been reported to occur without TSC [6, 9]. Notably, in these cases, multiple organs and TSC gene sequencing were not sufficiently assessed. Similarly, this case also lacked the relevant clinical manifestation of TSC, but this case had undergone genetic sequencing of TSC to confirm the diagnosis of TSC. To our best knowledge, this is the first case of MMPH associated atypical TSC confirmed by pathological biopsy combined with TSC gene detection.

Most patients with MMPH are asymptomatic, and the physiology or prognosis of MMPH is unclear, with the available literature suggesting an overall stable disease course [7, 10]. HRCT of MMPH is characterized by randomly distributed GGOs or solid nodules in both lungs, the size of which is approximately 2–10 mm [11]. In this case, chest CT showed diffuse ground-glass density nodules in the bilateral lung fields, particularly in the middle and upper lungs. During the follow-up, no significant change was found in multiple pulmonary nodules, suggesting that the radiological findings of MMPH patients may be stable. The histological feature of MMPH is that multicentric, well-defined type II alveolar epithelial cells grow modularly along the alveolar septum, characterized by thickening of fibers, an increase in elastic fibers and the accumulation of alveolar macrophages [4, 5]. Regarding histopathological manifestations, distinguishing MMPH from AAH and non-mucinous AIS is challenging. In most AHH and non-mucous AIS, no alveolar collapse, an increase in elastic fibers in the alveolar septum, the accumulation of tissue cells in the alveolar cavity, and a larger nucleus and cytoplasm of alveolar epithelial cells than in MMPH [4, 12]. MMPH was once described as an acinar atypical adenomatoid proliferation of the epithelium, also indicating the difficulty of its differential diagnosis. In this case, the pathology of lung biopsy revealed active hyperplasia of the alveolar epithelium, many tissue cells gathered in the alveolar cavity, and widening of the alveolar space was observed in some nodules, with fibrous tissue hyperplasia and scattered lymphocyte infiltration. In the immunohistochemical investigation, TTF-1 (+) and CD68 (tissue cells in the alveolar cavity++) alveolar epithelium and the positive rates of p53 and Ki67 were low. These manifestations were consistent with the histological and immunohistochemical performance of MMPH, but the clinical diagnosis must be considered with the medical history. However, the patient had neither a history of TSC nor symptoms of epilepsy, mental retardation, or sebaceous adenoma, which are typical clinical features of TSC. However, in combination with the patient's imaging findings, this patient met one major criterion (subcortical nodules) and one minor criterion (multiple renal cysts), considering the possibility of TSC from the perspective of the clinical diagnosis. To further clarify the diagnosis of TSC, further TSC gene testing was performed, and the result revealed a nonsense mutation in exon 9 of the TSC1 gene, further confirming the diagnosis of TSC and MMPH.

TSC is an autosomal-dominant neurocutaneous disease with high phenotypic variability and is characterized by classic Vogt’s triad of facial sebaceous adenoma, epilepsy, and mental retardation [11, 13]. The pathological changes of TSC are mainly multiple hamartoma lesions in many organs, most often involving the skin and central nervous system, as well as the kidney, lung, liver, heart, blood vessels and skeletal systems [14]. It is mainly a clinical diagnosis based on clinical features, but many cases had insufficient clinical characteristics in the disease diagnosis. With the development of genetic analysis technology, the diagnostic criteria updated by the International TSC Consensus Conference in 2012 added the results of genetic testing, considering the pathogenic mutation of TSC1 or TSC2 as an independent diagnostic criterion [2]. The TSC1 and TSC2 genes are considered tumor suppressor genes. TSC is caused by mutations in either TSC1 (chromosome 9q34) or TSC2 (chromosome 16p13) gene, which encode hamartin and tuberin, respectively [15]. A “pathogenic” mutation was defined as a mutation that clearly prevents protein synthesis and/or inactivates the function of the TSC1 or TSC2 proteins [2, 16]. Previous studies have shown that the pathogenic germline mutations of TSC1/TSC2 are mainly small insertion-deletion mutations or nonsense mutations [13]. In this case, gene sequencing suggested that the nonsense mutation of the TSC1 gene resulted in the loss of protein expression. According to related studies, pathogenic variants in TSC2 are usually associated with a more severe phenotype of TSC than TSC1, and TSC arising from a TSC1 mutation is less symptomatic and more likely to be overlooked [17, 18]. Inactivation mutation in TSC1 or TSC2 leads to hyperactivation of the mechanistic target of rapamycin (mTOR) pathway, which results in increased cell growth and proliferation in multiple organ systems, including the lung [17]. More than 50% of TSC cases are associated with lung involvement, and the prevalence increases with age [19]. Well-established pulmonary manifestations of TSC include lymphangioleiomyomatosis (LAM) and MMPH. MMPH is less common than LAM which is typical of cystic destruction with smooth muscle-like spindle cell infiltration and aggregation [20].

In conclusion, we describe an asymptomatic case of MMPH secondary to TSC diagnosed by pathological biopsy combined with genetic sequencing. Thus, MMPH must be considered in the differential diagnosis of multiple ground-glass nodules in both lungs, even in cases lacking clinical manifestations of TSC, and the diagnosis can be further assisted by imaging examination, histopathology and genetic sequencing.

## Data Availability

The datasets used during the current study are available from the corresponding author on reasonable request. The raw sequence data reported in this paper have been deposited in the Genome Sequence Archive (Genomics, Proteomics & Bioinformatics 2021) in National Genomics Data Center (Nucleic Acids Res 2021), China National Center for Bioinformation / Beijing Institute of Genomics, Chinese Academy of Sciences (GSA-Human Accession number: HRA001808) that are publicly accessible at https://ngdc.cncb.ac.cn/gsa-human.

## Abbreviations

MMPH: Multifocal micronodular pneumocyte hyperplasia
TSC: Tuberous sclerosis complex
LAM: Lymphangioleiomyomatosis
GGOs: Ground-glass opacities
AAH: Atypical adenomatous hyperplasia
AIS: Adenocarcinoma in situ
MIA: Minimally invasive adenocarcinoma
PET-CT: Positron emission computed tomography
NMD: Nonsense-mediated mRNA degradation
MRI: Magnetic resonance imaging
HRCT: High-resolution computed tomography
mTOR: Mechanistic target of rapamycin
